# Orthopedic Frailty Score and adverse outcomes in patients with surgically managed isolated traumatic spinal injury

**DOI:** 10.1136/tsaco-2023-001265

**Published:** 2024-07-11

**Authors:** Ahmad Mohammad Ismail, Frank Hildebrand, Maximilian Peter Forssten, Marcelo A F Ribeiro Jr., Parker Chang, Yang Cao, Babak Sarani, Shahin Mohseni

**Affiliations:** 1 School of Medical Sciences, Orebro University, Orebro, Sweden; 2 Department of Orthopedic Surgery, Orebro University Hospital, Orebro, Sweden; 3 Department of Orthopedics, Trauma, and Reconstructive Surgery, Uniklinik RWTH Aachen, Aachen, Germany; 4 Department of Surgery, Sheikh Shakhbout Medical City, Abu Dabi, UAE; 5 Pontifical Catholic University of Sao Paulo, Sao Paulo, Brazil; 6 Center for Trauma and Critical Care, The George Washington University, Washington, District of Columbia, USA; 7 Clinical Epidemiology and Biostatistics, School of Medical Sciences, Faculty of Medicine and Health, Örebro University, Orebro, Sweden

**Keywords:** frailty, mortality, complications, spine

## Abstract

**Background:**

With an aging global population, the prevalence of frailty in patients with traumatic spinal injury (TSI) is steadily increasing. The aim of the current study is to evaluate the utility of the Orthopedic Frailty Score (OFS) in assessing the risk of adverse outcomes in patients with isolated TSI requiring surgery, with the hypothesis that frailer patients suffer from a disproportionately increased risk of these outcomes.

**Methods:**

The Trauma Quality Improvement Program database was queried for all adult patients (18 years or older) who suffered an isolated TSI due to blunt force trauma, between 2013 and 2019, and underwent spine surgery. Patients were categorized as non-frail (OFS 0), pre-frail (OFS 1), or frail (OFS ≥2). The association between the OFS and in-hospital mortality, complications, and failure to rescue (FTR) was determined using Poisson regression models, adjusted for potential confounding.

**Results:**

A total of 43 768 patients were included in the current investigation. After adjusting for confounding, frailty was associated with a more than doubling in the risk of in-hospital mortality (adjusted incidence rate ratio (IRR) (95% CI): 2.53 (2.04 to 3.12), p<0.001), a 25% higher overall risk of complications (adjusted IRR (95% CI): 1.25 (1.02 to 1.54), p=0.032), a doubling in the risk of FTR (adjusted IRR (95% CI): 2.00 (1.39 to 2.90), p<0.001), and a 10% increase in the risk of intensive care unit admission (adjusted IRR (95% CI): 1.10 (1.04 to 1.15), p=0.004), compared with non-frail patients.

**Conclusion:**

The findings indicate that the OFS could be an effective method for identifying frail patients with TSIs who are at a disproportionate risk of adverse events.

**Level of evidence:**

Level III.

**WHAT IS ALREADY KNOWN ON THIS TOPIC**

- With an aging global population, the prevalence of frailty in patients with traumatic spinal injury is steadily increasing.

**WHAT THIS STUDY ADDS**

- The Orthopedic Frailty Score could be an effective method for identifying frail patients with traumatic spinal injuries who are at a disproportionate risk of adverse events.

**HOW THIS STUDY MIGHT AFFECT RESEARCH, PRACTICE OR POLICY**

- The Orthopedic Frailty Score is a simple and efficient tool that could help identify patients with traumatic spinal injury who require improved preoperative optimization, increased levels of postoperative monitoring, or other interventions that may potentially improve postoperative outcomes.

## Introduction

 Traumatic spinal injury (TSI) encompasses a range of damage to the spinal column, including the spinal cord, nerve roots, osseous structures, and discoligamentous components.^1^,^3^ The most common cause of TSI is blunt trauma, typically from motor vehicle accidents, followed by falls and sport injuries. The consequences of spinal column injury may include mechanical instability, impaired movements, and harm to the neural structures, which can result in partial or complete paralysis. A subset of TSI, known as traumatic spinal cord injury, is characterized by neurological deficits subsequent to mechanical forces experienced by the spinal cord.^1^,^5^ Historically, TSI was more prevalent among men in their 40s, but with an aging global population, the average age of those affected by TSI continues to rise.^15^,^8^ It is predicted that a significant proportion of new TSI cases will occur in older patients, a group who is particularly at risk of adverse events after injury due to the higher prevalence of frailty and other medical conditions.^59^,^14^

Although timely surgical intervention has been shown to lower morbidity and mortality,^15^,^17^ patients who undergo spinal surgery after trauma are at risk of postoperative complications and death.^5 11 14 18^ Healthcare providers might improve patient outcomes by identifying frailty and prioritizing those who are more susceptible to adverse outcomes for prompt, comprehensive and multidisciplinary management.^18 19^ This approach might also aid in the efforts to optimize resource utilization for maximum benefit and ensure the best possible outcomes for this vulnerable patient population.^19^

Frailty is a syndrome marked by decreased physiologic reserve and increased vulnerability to external stressors. This condition is more prevalent in older individuals and various tools have been proposed to characterize and measure it.^11 18 20^ The latest systematic review on frailty in TSIs found that frailty, regardless of the specific index or measure employed, is associated with a heightened risk of morbidity and mortality in patients undergoing spine surgery.^18^ Nonetheless, many of these indices are impractical to use in the clinical setting or otherwise fail to adequately capture frailty, due to primarily measuring comorbidity burden or fitness for surgery. The Orthopedic Frailty Score (OFS), a novel frailty score that has been proposed to measure frailty using only five dichotomous variables, has shown promising results when applied to patients with traumatic hip fracture.^21 22^ The aim of the current study is to evaluate the utility of the OFS in assessing the risk of adverse outcomes in patients with TSI, with the hypothesis that frailer patients suffer from a disproportionately increased risk of these outcomes.

## Methods

All information necessitated by the subsequent analyses was appropriated from the American College of Surgeons Trauma Quality Improvement Program (TQIP) database. Data retrieved included: age, sex, race, the Abbreviated Injury Scale (AIS) score for each body region, comorbidities, presence of an advanced directive limiting care, injury characteristics, type of surgery, discharge disposition, and complications. Patients registered in TQIP between 2013 and 2019 were considered for inclusion if they were an adult (18 years or older) who suffered an isolated TSI as a consequence of a blunt force trauma, which required surgical intervention. An isolated TSI was defined as a spine AIS score of ≥2 and an AIS score of ≤1 in the remaining regions. Patients were excluded if they had a missing OFS or if they had an AIS score of 6 in any region.

All aspects of the current investigation comply with the Declaration of Helsinki as well as the Strengthening the Reporting of Observational Studies in Epidemiology guidelines.^23^

### Calculating the OFS

The OFS is a recently validated score that was originally developed for measuring frailty and predicting short-term mortality in patients with hip fracture. The OFS was calculated based on the five dichotomous variables: congestive heart failure, a history of malignancy (local or metastatic, excluding non-invasive skin cancer), institutionalization (admitted from a nursing home, long-term care facility, or other group living arrangement), non-independent functional status (ie, requiring assistance with activities of daily life), and an age ≥85 years old. A patient received 1 point for each variable present, with the maximum possible score being 5. Patients with an OFS ≥2 were considered frail, according to the original study.^21 22^

### Statistical analysis

Patients were separated into cohorts based on their OFS: non-frail (OFS 0), pre-frail (OFS 1), and frail (OFS ≥2).^22^ As a consequence of continuous variables being non-normally distributed, they were summarized as medians and IQRs, whereas categorical variables were instead organized as counts and percentages. The Kruskal-Wallis test was employed to evaluate the statistical significance of differences between continuous variables and either the χ^2^ test or Fisher’s exact test was applied, as appropriate, to categorical variables. The primary outcome of interest was in-hospital mortality, with the secondary outcomes consisting of any in-hospital complication (myocardial infarction, cardiac arrest with cardiopulmonary resuscitation, stroke, deep vein thrombosis, pulmonary embolism, acute respiratory distress syndrome, pneumonia, or surgical site infection), failure to rescue (FTR), as well as intensive care unit (ICU) admission. FTR was defined as in-hospital mortality after a complication.

Poisson regression models were used to determine the association between frailty and the previously mentioned adverse outcomes, adjusted for covariates available to minimize potential confounding. When constructing the models, in-hospital mortality, complications, or FTR was set as the response variable, whereas the explanatory variables consisted of frailty (measured using the OFS), as well as age, sex, race, highest AIS in each region, level of injury, presence of spinal cord injury, level of spine surgery, comorbidities (hypertension, previous myocardial infarction, history of peripheral vascular disease, cerebrovascular disease, dementia, chronic obstructive pulmonary disease, smoking status, chronic renal failure, diabetes mellitus, cirrhosis, coagulopathy, drug use disorder, alcohol use disorder, major psychiatric illness), and advanced directives limiting care. Due to the generally short length of stay, no correction was required for time dependence in the Poisson regression analysis. Results are presented as adjusted incidence rate ratios (IRRs) and corresponding 95% CIs were calculated with robust SEs.

A two-sided p value less than 0.05 was considered statistically significant. Among patients included in the current dataset, <2% had any form of missing data. Subsequently, data that were missing were assumed to be missing at random. To manage missing data, multiple imputation by chained equations was employed; logistic regression was used for sex and race whereas a proportional odds model for the spine AIS. Analyses were performed using the *tidyverse, haven, mice,* and *sandwich* packages in the statistical software R V.4.2.2 (R Foundation for Statistical Computing, Vienna, Austria).^24^

## Results

After applying the listed inclusion and exclusion criteria, a total of 43 768 patients remained for further analysis. According to their OFS, 87.3% were non-frail (N=38 237), 10.1% were pre-fail (N=4411) and 2.6% were frail (N=1120). Pre-frail and frail patients were generally older (76 and 85 vs. 53 years old, p<0.001), more likely to be female (37.7% and 42.5% vs. 31.1%, p<0.001), more likely to have advanced directives limiting care (6.8% and 15.7% vs. 1.3%, p<0.001), and exhibited a higher prevalence of almost all comorbidities, compared with non-frail patients. The only conditions that decreased in prevalence in frailer patients were active smoker, drug use disorder, and alcohol use disorder (table 1). Frail patients were also less severely injured on average compared with non-frail patients (spine AIS ≥4: 17.2% vs. 26.1%, p<0.001). Consequently, although frail patients were more likely to have a cervical spine injury compared with non-frail patients (64.1% vs. 60.6%, p<0.001), they were in fact less likely to have suffered a cervical *spinal cord* injury (23.1% vs. 31.9%, p<0.001) (table 2).

**Table 1:** Demographics of patients with surgically managed isolated traumatic spine injuries

	Non-frail (N=38 237)	Pre-frail (N=4411)	Frail (N=1120)	P value
Age, median (IQR)	53 (36–66)	76 (65–85)	85 (76–87)	<0.001
Sex, n (%)				<0.001
Female	11 880 (31.1)	1661 (37.7)	476 (42.5)	
Male	26 347 (68.9)	2748 (62.3)	644 (57.5)	
Missing	10 (0.0)	2 (0.0)	0 (0.0)	
Race, n (%)				
White	29 278 (76.6)	3652 (82.8)	999 (89.2)	<0.001
Black	4589 (12.0)	432 (9.8)	59 (5.3)	<0.001
Asian	830 (2.2)	103 (2.3)	16 (1.4)	0.178
American Indian	349 (0.9)	15 (0.3)	1 (0.1)	<0.001
Pacific Islander	109 (0.3)	11 (0.2)	1 (0.1)	0.562
Other	2429 (6.4)	147 (3.3)	25 (2.2)	<0.001
Missing	422 (1.1)	31 (0.7)	9 (0.8)	
Hypertension, n (%)	12 914 (33.8)	2989 (67.8)	849 (75.8)	<0.001
Previous myocardial infarction, n (%)	287 (0.8)	85 (1.9)	36 (3.2)	<0.001
Congestive heart failure, n (%)	0 (0.0)	1005 (22.8)	523 (46.7)	<0.001
History of peripheral vascular disease, n (%)	177 (0.5)	81 (1.8)	39 (3.5)	<0.001
Cerebrovascular disease, n (%)	519 (1.4)	290 (6.6)	85 (7.6)	<0.001
Dementia, n (%)	469 (1.2)	401 (9.1)	246 (22.0)	<0.001
Non-independent functional status, n (%)	0 (0.0)	1531 (34.7)	768 (68.6)	<0.001
Institutionalized, n (%)	0 (0.0)	430 (9.7)	420 (37.5)	<0.001
History of malignancy, n (%)	0 (0.0)	282 (6.4)	81 (7.2)	<0.001
COPD, n (%)	2016 (5.3)	655 (14.8)	196 (17.5)	<0.001
Current smoker, n (%)	9726 (25.4)	574 (13.0)	61 (5.4)	<0.001
Chronic renal failure, n (%)	307 (0.8)	210 (4.8)	66 (5.9)	<0.001
Diabetes mellitus, n (%)	5659 (14.8)	1469 (33.3)	403 (36.0)	<0.001
Cirrhosis, n (%)	302 (0.8)	57 (1.3)	19 (1.7)	<0.001
Coagulopathy, n (%)	951 (2.5)	360 (8.2)	144 (12.9)	<0.001
Drug use disorder, n (%)	2623 (6.9)	156 (3.5)	14 (1.3)	<0.001
Alcohol use disorder, n (%)	3409 (8.9)	298 (6.8)	31 (2.8)	<0.001
Major psychiatric illness, n (%)	3785 (9.9)	700 (15.9)	208 (18.6)	<0.001
Advanced directive limiting care, n (%)	511 (1.3)	302 (6.8)	176 (15.7)	<0.001

Non-frail, pre-frail, and frail are defined as OFS 0, OFS 1, and OFS ≥2.

COPDchronic obstructive pulmonary diseaseOFSOrthopedic Frailty Score

**Table 2:** Clinical characteristics of patients with surgically managed isolated traumatic spine injuries

	Non-frail (N=38 237)	Pre-frail (N=4411)	Frail (N=1120)	P value
Head AIS, n (%)				0.070
Injury not present	32 945 (86.2)	3748 (85.0)	954 (85.2)	
1	5292 (13.8)	663 (15.0)	166 (14.8)	
Face AIS, n (%)				<0.001
Injury not present	31 751 (83.0)	3505 (79.5)	871 (77.8)	
1	6486 (17.0)	906 (20.5)	249 (22.2)	
Neck AIS, n (%)				0.438
Injury not present	37 726 (98.7)	4351 (98.6)	1100 (98.2)	
1	511 (1.3)	60 (1.4)	20 (1.8)	
Spine AIS, n (%)				<0.001
2	14 198 (37.1)	1635 (37.1)	465 (41.5)	
3	14 072 (36.8)	1723 (39.1)	462 (41.2)	
4	7248 (19.0)	808 (18.3)	160 (14.3)	
5	2703 (7.1)	241 (5.5)	33 (2.9)	
Missing	16 (0.0)	4 (0.1)	0 (0.0)	
Thorax AIS, n (%)				0.428
Injury not present	35 902 (93.9)	4158 (94.3)	1059 (94.6)	
1	2335 (6.1)	253 (5.7)	61 (5.4)	
Abdomen AIS, n (%)				<0.001
Injury not present	37 120 (97.1)	4324 (98.0)	1104 (98.6)	
1	1117 (2.9)	87 (2.0)	16 (1.4)	
Upper extremity AIS, n (%)				0.365
Injury not present	34 206 (89.5)	3924 (89.0)	991 (88.5)	
1	4031 (10.5)	487 (11.0)	129 (11.5)	
Lower extremity AIS, n (%)				0.208
Injury not present	34 535 (90.3)	4017 (91.1)	1020 (91.1)	
1	3702 (9.7)	394 (8.9)	100 (8.9)	
External/other AIS, n (%)				<0.001
Injury not present	36 567 (95.6)	4269 (96.8)	1084 (96.8)	
1	1670 (4.4)	142 (3.2)	36 (3.2)	
Level of spine injury, n (%)				
Cervical	23 171 (60.6)	2934 (66.5)	718 (64.1)	<0.001
Thoracic	10 492 (27.4)	1230 (27.9)	338 (30.2)	0.113
Lumbar	10 904 (28.5)	797 (18.1)	209 (18.7)	<0.001
Spinal cord injury, n (%)				
Cervical	12 210 (31.9)	1367 (31.0)	259 (23.1)	<0.001
Thoracic	2150 (5.6)	239 (5.4)	51 (4.6)	0.274
Lumbar	1892 (4.9)	87 (2.0)	26 (2.3)	<0.001
Level of spine surgery, n (%)				
Cervical	27 809 (72.7)	3311 (75.1)	794 (70.9)	0.001
Thoracic	21 062 (55.1)	2245 (50.9)	559 (49.9)	<0.001
Lumbar	17 922 (46.9)	1645 (37.3)	389 (34.7)	<0.001

Non-frail, pre-frail, and frail are defined as OFS 0, OFS 1, and OFS ≥2.

AISAbbreviated Injury ScaleOFSOrthopedic Frailty Score

Prior to adjustment, both pre-frail and frail patients exhibited higher rates of in-hospital mortality (6.1% and 11.9% vs. 1.5%, p<0.001), complications (9.7% and 9.0% vs. 5.2%, p<0.001), FTR (2.6% and 3.8% vs. 0.7%, p<0.001), and ICU admission (63.0% and 62.1% vs. 54.9%, p<0.001), compared with non-frail patients. The majority of the specific complications measured were also more prevalent among pre-frail and frail patients (table 3). After adjusting for confounding, frailty was associated with a more than doubling in the risk of in-hospital mortality (adjusted IRR (95% CI): 2.53 (2.04 to 3.12), p<0.001), a 25% higher overall risk of complications (adjusted IRR (95% CI): 1.25 (1.02 to 1.54), p=0.032), a doubling in the risk of FTR (adjusted IRR (95% CI): 2.00 (1.39 to 2.90), p<0.001), and a 10% increase in the risk of ICU admission (adjusted IRR (95% CI): 1.10 (1.04 to 1.15), p=0.004), compared with non-frail patients (table 4).

**Table 3:** Crude outcomes in patients with surgically managed isolated traumatic spine injuries

	Non-frail (N=38 237)	Pre-frail (N=4411)	Frail (N=1120)	P value
In-hospital mortality, n (%)	562 (1.5)	271 (6.1)	133 (11.9)	<0.001
Any complication, n (%)	2001 (5.2)	429 (9.7)	101 (9.0)	<0.001
Myocardial infarction, n (%)	69 (0.2)	28 (0.6)	15 (1.3)	<0.001
Cardiac arrest with CPR, n (%)	366 (1.0)	96 (2.2)	25 (2.2)	<0.001
Stroke, n (%)	69 (0.2)	22 (0.5)	8 (0.7)	<0.001
DVT, n (%)	551 (1.4)	112 (2.5)	19 (1.7)	<0.001
Pulmonary embolism, n (%)	274 (0.7)	41 (0.9)	7 (0.6)	0.266
ARDS, n (%)	252 (0.7)	41 (0.9)	10 (0.9)	0.087
Pneumonia, n (%)	811 (2.1)	141 (3.2)	36 (3.2)	<0.001
Surgical site infection, n (%)	118 (0.3)	28 (0.6)	7 (0.6)	<0.001
Failure to rescue, n (%)	259 (0.7)	114 (2.6)	43 (3.8)	<0.001
ICU admission, n (%)	20 993 (54.9)	2781 (63.0)	695 (62.1)	<0.001

Non-frail, pre-frail, and frail are defined as OFS 0, OFS 1, and OFS ≥2.

ARDSacute respiratory distress syndromeCPRcardiopulmonary resuscitationDVTdeep vein thrombosisICUintensive care unitOFSOrthopedic Frailty Score

**Table 4:** IRRs for adverse outcomes in patients with surgically managed isolated traumatic spine injuries

Adverse outcome	Non-frail	Pre-frail IRR (95% CI)	P value	Frail IRR (95% CI)	P value
In-hospital mortality	Reference	1.62 (1.38 to 1.90)	<0.001	2.53 (2.04 to 3.12)	<0.001
Any complication	Reference	1.33 (1.19 to 1.49)	<0.001	1.25 (1.02 to 1.54)	0.032
Failure to rescue	Reference	1.59 (1.24 to 2.04)	<0.001	2.00 (1.39 to 2.90)	<0.001
ICU admission	Reference	1.09 (1.06 to 1.12)	<0.001	1.10 (1.04 to 1.15)	0.004

Non-frail, pre-frail, and frail are defined as OFS 0, OFS 1, and OFS ≥2.

IRRs are calculated using Poisson regression models with robust SEs. Missing values were managed using multiple imputation by chained equations. All analyses were adjusted for age, sex, race, highest Abbreviated Injury Scale in each region, level of injury, presence of spinal cord injury, level of spine surgery, hypertension, previous myocardial infarction, history of peripheral vascular disease, cerebrovascular disease, dementia, chronic obstructive pulmonary disease, smoking status, chronic renal failure, diabetes mellitus, cirrhosis, coagulopathy, drug use disorder, alcohol use disorder, major psychiatric illness, and advanced directives limiting care.

ICUintensive care unitIRRsincidence rate ratiosOFSOrthopedic Frailty Score

## Discussion

The world is undergoing a demographic shift with an increasing proportion of elderly individuals in the population, a trend attributed to factors such as advances in medical technology and improved living conditions. In developed countries, the elderly population is growing faster than the general population, and this trend is expected to continue.^25^,^27^ This shift is also reflected in the population affected by trauma and subsequent TSIs.^15^,^14^ Historically, TSI was most commonly seen in men in their 40s; however, TSIs are now becoming more prevalent in older patients who are more vulnerable to adverse outcomes often due to underlying frailty or comorbidities.^59^,^14^ It is crucial for healthcare providers to confront this challenge to provide adequate support and resources for this ever-growing population who is now being affected by TSI in higher rates.

In view of the limited nature of healthcare resources, particularly in universal healthcare systems where the demand for these resources exceeds the supply, the proper allocation of resources such as personnel, operating rooms, equipment, and access to high levels of care such as ICUs, presents a significant challenge and is of utmost importance.^28^,^30^ Hence, it is imperative that effective approaches are adopted to efficiently allocate resources and provide optimal care to all patients. One potential strategy could be to identify frail patients who may require the most resources and additional intervention early on, for example, improved preoperative optimization, expedited surgery, additional or extended prophylaxis against postoperative adverse outcomes, increased levels of postoperative monitoring, as well as orthogeriatric care, to implement targeted strategies that could mitigate the most costly adverse events.

Frailty is a clinical state where patients exhibit a lower physiological capacity to handle external stressors due to a deterioration in multiple organ systems. This results in a heightened likelihood of suffering from postoperative complications and mortality.^5 18^ Although frailty is often considered a hallmark of aging, it is important to note that it is an independent process and not an inevitable outcome of aging.

In the current study, we could demonstrate that patients who were considered frail, according to the OFS, were at a higher risk of overall complications, ICU admission, FTR, as well as in-hospital mortality, compared with non-frail patients. These results are in line with previous studies that investigate the association between frailty and postoperative outcomes in patients with TSI.^5 11 18^ According to a 2020 systematic review conducted by Chan *et al*, which included 32 studies conducted between 1946 and 2020, eight different frailty indices or measures were used during this time period. The frailty indices that were analyzed in the studies include the 11-point modified Frailty Index (mFI-11), Adult Spinal Deformity Frailty Index (ASDFI), psoas size, Metastatic Spine Tumor Frailty Index, Cervical Deformity Frailty Index, 5-item modified Frailty Index (mFI-5), Hospital Frailty Risk Score, and the FRAIL Scale. Of these, mFI-11 and ASDFI were the most frequently studied and the results of the review indicated that all of these frailty indices or measures were associated with an increased risk of postoperative morbidity and mortality.^18^ The largest study included in the systematic review was a multicenter, ambispective study that analyzed 52 671 patients and used the mFI-11. This study showed that a higher mFI-11 score was associated with an increased risk of adverse events (OR 1.58 (1.51 to 1.64) per 0.10 increase in the mFI score) and mortality (OR 2.05 (1.69 to 2.47)).^31^ Additionally, a recent article published in 2022 found that the Risk Analysis Index and its recalibrated version were superior in predicting postoperative outcomes compared with the mFI-5 in TSI.^5^ Finally, although the Trauma-Specific Frailty Index has not yet been tested specifically in patients with TSI,^32^ it has been found in the geriatric trauma population to be significantly associated with an increased risk of mortality, complications, unfavorable discharge, readmission, and fall recurrence.^33^ Nevertheless, a challenge with the previously studied frailty indices or measures is their difficult to implement in clinical practice due to time-consuming and at times not readily available data at the time of admission. The OFS, which is based on five variables that are easily obtained upon arrival in the emergency room, offers a practical solution. However, there are some limitations that are important to take into consideration.

The OFS was initially developed using a cohort of patients with hip fractures and not TSI. Nonetheless, the findings of this study, along with other research, suggest this frailty score is also applicable to patients with TSI.^20^ This is not surprising given the similarities between these two populations in terms of age, comorbidity burden, and degree of frailty. Patients with TSI, however, are a diverse patient population with varying prognoses based on factors such as the type of injury and existing comorbidities, with subgroups such as patients with ankylosing spondylitis and diffuse idiopathic skeletal hyperostosis presenting unique challenges.^11 12 18^ The current study did not account for these variables. Additionally, data on the impact of frailty on health-related quality of life outcomes were not available. These limitations are primarily a result of the study’s retrospective design. Furthermore, the presence of other weaknesses of retrospective study designs cannot be completely eliminated, such as the possibility of residual confounding, the reliance on the accuracy of data recorded in the dataset, and the unavailability of other variables not contained in the dataset, including the cause of death, preoperative optimization, and intraoperative factors. Despite these limitations, the present study possesses several advantages, one of which being the utilization of a relatively extensive national sample population comprising patients who received treatment at more than 875 trauma centers throughout the USA. Due to the wide range of information capture by this dataset, adjustments could be made to account for differences in a substantial number of preadmission comorbidities as well as racial and demographic variations.

## Conclusion

The findings indicate that the OFS could be an effective method for identifying frailty in patients with TSI who are at a disproportionate risk of adverse postoperative events.

## Data Availability

Data may be obtained from a third party and are not publicly available.
